# Pathway-specific regulation of the paraventricular thalamic nucleus in depressive-like behavior

**DOI:** 10.1038/s41598-026-45354-y

**Published:** 2026-04-02

**Authors:** Momoe Kassai, Daiki Tachibana, Fumiaki Sato, Hajime Miyanishi, Haruna Yoshida, Taro Kato, Mie Sakashita-Kubota, Tadafumi Kato, Yasuyuki Shima

**Affiliations:** 1 Department of Psychiatry and Behavioral Science, Juntendo University Graduate School of Medicine, 2-1-1 Hongo, Bunkyo-ku, Tokyo 113-8421 Japan; 2 Sumitomo Pharma Co., Ltd., Osaka, Japan; 3 Department of Molecular Pathology of Mood Disorders, Juntendo University Graduate School of Medicine, Tokyo, Japan; 4 Neurodegenerative Disorders Collaborative Laboratory, RIKEN Center for Brain Science, Hirosawa 2-1, Wako, Saitama 351-0198 Japan

**Keywords:** Diseases, Genetics, Neuroscience

## Abstract

Bipolar disorder is one of major psychiatric disorders afflicting about 1% of the world population. While genetic factors have been increasingly recognized as contributing to the disorder, the underlying etiology of bipolar disorder, such as associated brain regions and cellular pathology, are yet to be elucidated. Our previous studies using a genetic bipolar disorder animal model indicated potential role of the paraventricular thalamic nucleus (PVT) in recurrent depressive-like episodes that exhibit phenotypic similarity to the depression phase of bipolar disorder. Notably, chronic activation of PVT induced spontaneous depressive-like episodes. Primary projection targets of PVT include the nucleus accumbens (NAc) and basolateral amygdala (BLA). However, the circuit involving PVT that causes recurrent depressive-like episodes remains to be addressed. Thus, we activated the PVT neurons projecting to NAc or BLA selectively to investigate their roles in eliciting depressive-like episodes. We found that activation of the PVT–NAc pathway induced depressive-like episodes, as measured by reduced wheel running activity in mice. In contrast, wheel running activity was unaffected by activation of the PVT–BLA pathway. These results highlight the critical involvement of PVT–NAc circuit in mood regulation and suggest it may be a key neural substrate underlying depressive-like episodes in bipolar disorder.

## Introduction

Paraventricular nucleus of thalamus (PVT) is increasingly recognized as a pivotal hub mediating motivation-related behaviors^1–5^. PVT is one of midline thalamic nuclei and receives diverse inputs from mood-related brain areas, such as serotonin neurons in the dorsal raphe, noradrenergic neurons in the locus coeruleus, medial prefrontal cortex, and several hypothalamic neurons^6–9^. It projects to several key brain regions implicated in emotion, including the nucleus accumbens (NAc), amygdala, and medial prefrontal cortex. Notably, the two primary PVT outputs—PVT to nucleus accumbens (PVT–NAc) and PVT to basolateral amygdala (PVT-BLA)—regulate a range of anxiety- and motivation-related behaviors such as conditioned fear, drug withdrawal, food intake, and wakefulness^4,5,10–14^.

Our previous studies using a genetic model mouse have shown possible involvement of PVT to expression of spontaneous, repetitive depressive-like episodes, which is unique characteristics of bipolar disorder. Multiple reports showed that mitochondria disease patients exhibit a much higher (~ 20%) rate of bipolar disorder^15–18^. From these observations, we generated a mouse line with neural expression of mutated mitochondria DNA polymerase (*Polg*)^19^, which is responsible for one of mitochondrial diseases, chronic progressive external ophthalmoplegia. Females of the transgenic mice (hereafter Polg Tg mice) exhibit spontaneous, recurrent episodes resembling depression^20^. Importantly, the number of depressive-like episodes is suppressed by administrating a mood stabilizer, lithium, suggesting that these mice could be a valid model bipolar disease. We further found that Polg Tg mice had mitochondrial DNA deletion particularly in the paraventricular thalamic nucleus (PVT) region, implying the involvement of the PVT in the depressive-like episodes. Our prior research demonstrated that prolonged activation of the PVT induced depressive-like episodes^21^, supporting the PVT’s significant role in depressive behaviors. However, it remains unclear which specific neural circuits—PVT–NAc, PVT–BLA, or others—contribute to these depressive-like episodes in the context of wheel running.

In this study, we employed chemogenetic technology using designer receptors exclusively activated by designer drugs (DREADDs) to dissect the roles of PVT–NAc or PVT–BLA pathway in inducing depressive-like episodes in female wild type mice. First, we assessed the off-target effects of the DREADD agonist, compound 21 (C21)^22^ and its impact on general locomotion. For projection target-specific expression of DREADD receptors, we infected Cre-dependent adeno-associated virus (AAV) for hM3Dq to PVT and AAV carrying retrograde (CAG)-nCre to one of the targets. Subsequently, we examined the effects of activating PVT neurons projecting to either the NAc or BLA on wheel running activity to identify the neural circuits responsible for depressive-like episodes.

## Results

### Pharmacological profile of DREADD agonist C21

Clozapine-N-oxide (CNO) is widely recognized for its robust activity as an agonist of DREADD receptor and is commonly used for short-term behavioral experiments. However, it has been reported that CNO can be converted to clozapine in vivo^23^, which may confound interpretation of long-term behavior studies. The DREADD agonist C21 is an alternative hM3Dq agonist with higher affinity (EC_50_ = 1.7 nM) than CNO (EC_50_ = 6.0 nM) and penetrates efficiently into the brain without converting to CNO nor clozapine^22,24^. Given these attributes, we evaluated C21 as an alternative to CNO and investigated whether it is suitable for long-term behavioral evaluation.

**Fig. 1:**
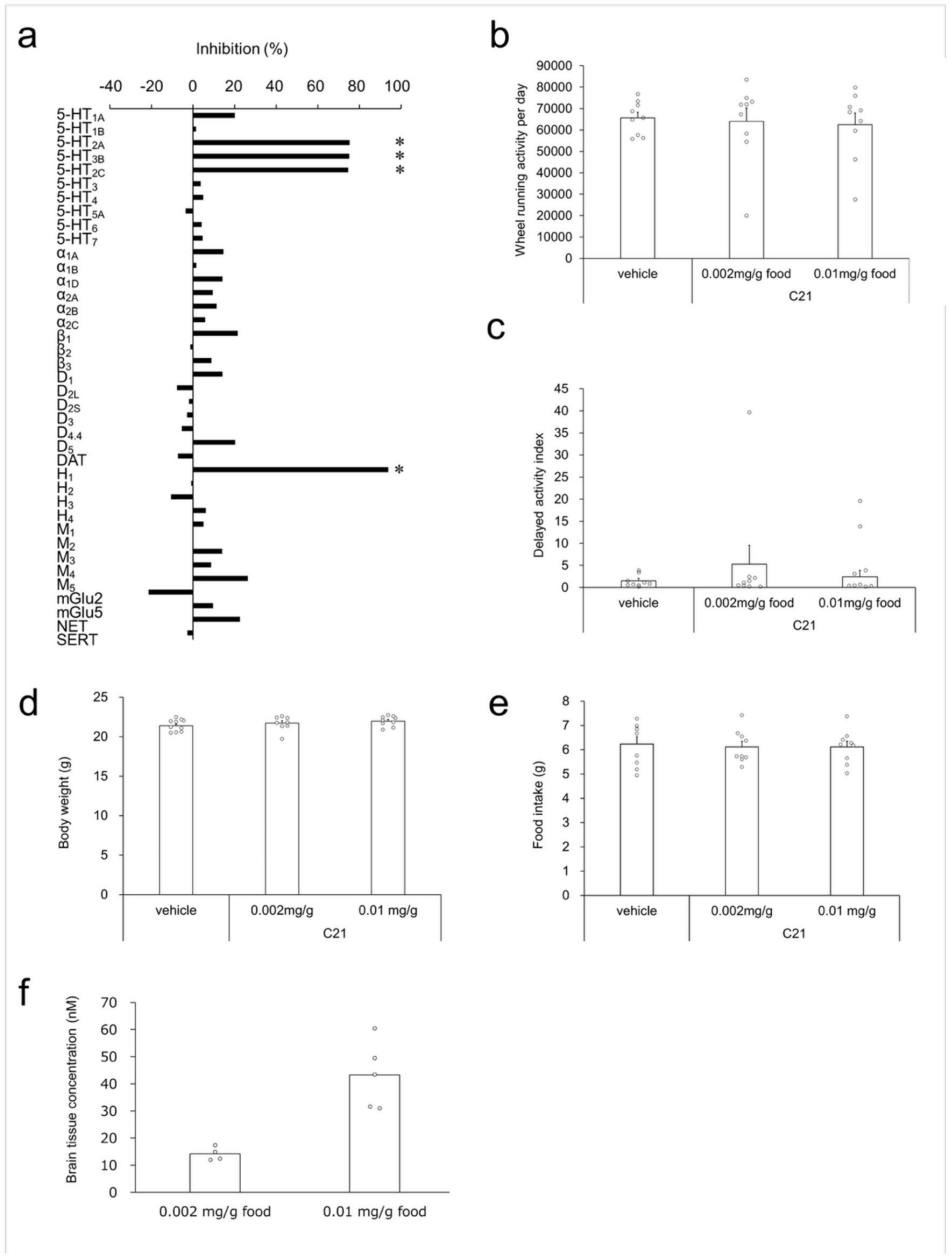
Assessment of Off-Target Binding and Impact of C21 on Wheel-Running Activity. (**a**) Off-target binding affinities of the C21 at a concentration of 100 nM for membranes expressing receptors, channels and transporters. The values and abbreviations are shown in Table 1. (**b**) Influence of C21 at 0.002 and 0.01 mg/g of food on wheel-running activity. Each bar represents the mean + S.E.M. of daily wheel-running activity over a 3-wk period (*n* = 9). No significant differences were observed in the wheel-running activity of mice administered C21 at different concentrations compared to those given the vehicle. (**c**) Effect of C21 at 0.002 and 0.01 mg/g of food on delayed activity index on wheel running. Each bar represents the mean + S.E.M. of delayed activity index over a 3-wk period (*n* = 9). (**d**) Effect of C21 at 0.002 and 0.01 mg/g of food on body weight. Each circle indicates body weight of each mouse, and each bar represents the mean + S.E.M. of body weight after wheel-running activity for 2 weeks (*n* = 9 each). (**e**) Effect of C21 on food intake on wheel running. Each circle indicates food intake of each mouse, and each bar represents the mean + S.E.M. of food intake over a 2-wk period (*n* = 9 each). (**f**) Concentration (nM) of C21 in the forebrain after wheel-running activity measurement for 3 weeks (0.002 mg/g food group: *n* = 4; 0.01 mg/g food group: *n* = 5). Each bar represents the mean of concentration of C21. Each circle indicates the C21 concentration of each mouse.

To assess its potential off-target activities of C21, we screened C21 at 100 nM against a panel of receptors. C21 showed greater than 50% inhibition of binding at several targets including histamine H_1_, serotonin 5 HT_2A_, 5 HT_2B_ and 5 HT_2C_ receptors (Fig. 1a, Table 1), indicating those receptors could be potential off-targets.

**Table 1:** Off-target binding profile of C21 at 100 nM.

Target	Radioligand	Radioligand conc. (nM)	% Inhibition
Human Serotonin (5-Hydroxytryptamine) 5-HT_1A_	[^3^H] 8-OH-DPAT	1.5	20.1
Human Serotonin (5-Hydroxytryptamine) 5-HT_1B_	[^3^H] GR125743	1	1.5
Human Serotonin (5-Hydroxytryptamine) 5-HT_2A_	[^3^H] Ketanserin	0.5	75.3
Human Serotonin (5-Hydroxytryptamine) 5-HT_2B_	[^3^H] Lysergic acid diethylamide	1.2	75.2
Human Serotonin (5-Hydroxytryptamine) 5-HT_2C_	[^3^H] Mesulergine	1	74.5
Human Serotonin (5-Hydroxytryptamine) 5-HT_3_	[^3^H] GR-65630	0.69	3.7
Guinea pig Serotonin (5-Hydroxytryptamine) 5-HT_4_	[^3^H] GR-113808	0.7	4.9
Human Serotonin (5-Hydroxytryptamine) 5-HT_5A_	[^3^H] Lysergic acid diethylamide	1.7	− 3.5
Human Serotonin (5-Hydroxytryptamine) 5-HT_6_	[^3^H] Lysergic acid diethylamide	1.5	4.2
Human Serotonin (5-Hydroxytryptamine) 5-HT_7_	[^3^H] Lysergic acid diethylamide	5.5	4.6
Human Adrenergic α_1A_	[^3^H] Prazosin	0.6	14.6
Human Adrenergic α_1B_	[^3^H] Prazosin	0.2	1.6
Human Adrenergic α_1D_	[^3^H] Prazosin	0.6	14.3
Human Adrenergic α_2A_	[^3^H] Rauwolscine	1.5	9.4
Human Adrenergic α_2B_	[^3^H] Rauwolscine	2.5	11.3
Human Adrenergic α_2C_	[^3^H] Rauwolscine	0.5	5.8
Human Adrenergic β_1_	[^125^I] Cyanopindolol	0.03	21.5
Human Adrenergic β_2_	[^3^H] CGP-12177	0.2	− 1.3
Human Adrenergic β_3_	[^125^I] Cyanopindolol	0.5	8.9
Human Dopamine D_1_	[^3^H] SCH-23390	1.4	14.2
Human Dopamine D_2L_	[^3^H] Spiperone	0.16	− 7.6
Human Dopamine D_2S_	[^3^H] Spiperone	0.16	− 2.0
Human Dopamine D_3_	[^3^H] Spiperone	0.7	− 2.8
Human Dopamine D_4.4_	[^3^H] Spiperone	1.2	− 5.4
Human Dopamine D_5_	[^3^H] SCH-23390	2	20.3
Human Transporter, Dopamine (DAT)	[^125^I] RTI-55	0.15	− 7.3
Human Histamine H_1_	[^3^H] Pyrilamine	1.2	93.9
Human Histamine H_2_	[^125^I] Aminopotentidine	0.1	− 0.9
Human Histamine H_3_	[^3^H] N-α-Methylhistamine	0.4	− 10.5
Human Histamine H_4_	[^3^H]-Histamine	10	6.2
Human Muscarinic M_1_	[^3^H] N-Methylscopolamine	0.8	5.1
Human Muscarinic M_2_	[^3^H] N-Methylscopolamine	0.8	0.0
Human Muscarinic M_3_	[^3^H] N-Methylscopolamine	0.8	14.1
Human Muscarinic M_4_	[^3^H] N-Methylscopolamine	0.8	8.7
Human Muscarinic M_5_	[^3^H] N-Methylscopolamine	0.8	26.3
Human Glutamate, Metabotropic, mGlu2	[3H]LY341495	2	− 21.4
Human Glutamate, Metabotropic, mGlu5	[^3^H] Quisqualic acid	0.03	9.6
Human Transporter, Norepinephrine (NET)	[^125^I] RTI-55	0.2	22.6
Human Transporter, Serotonin (5-Hydroxytryptamine) (SERT)	[^3^H] Paroxetine	0.4	− 2.6

Next, we tested the impact of C21 administration on long-term wheel running activity. We administrated C21 or vehicle to female mice for 3 weeks while monitoring their voluntary wheel-running activity. Dosages of 0.002 and 0.01 mg/g food showed no significant change of wheel running activity (vehicle: 65,699 ± 2578; 0.002 mg/g: 64,043 ± 6213; 0.01 mg/g: 62,465 ± 5430; *p* = 0.901; values are presented as mean ± standard error of the mean [S.E.M.]) (Fig. 1b). In addition to depressive-like episodes, the Polg Tg mouse shows a diurnal activity phenotype with prolonged wheel running in early daylight periods^19^. We examined their delayed activity indices, a measurement of the running activities in early light phases (see “Methods” section), and found that there was no significant difference (vehicle: 1.52 ± 0.51; 0.002 mg/g: 5.25 ± 4.31; 0.01 mg/g: 2.41 ± 1.48; *p* = 0.588) (Fig. 1c). Furthermore, we examined the body weight at 2 weeks after C21 administration, as well as the food intake during the 2‑week treatment period. Dosages of 0.002 and 0.01 mg/g food showed no significant change of body weight (vehicle: 21.37 ± 0.25; 0.002 mg/g: 21.73 ± 0.29; 0.01 mg/g: 21.97 ± 0.22 g; *p* = 0.262) and food intake (vehicle: 6.24 ± 0.30; 0.002 mg/g: 6.12 ± 0.23; 0.01 mg/g: 6.12 ± 0.23; *p* = 0.933, Fig. 1d, e). These results indicated that C21 of these dosages had no effect on locomotion activity, sleep–wake rhythm, body weight and food intake in wheel running.

Following the wheel running behavioral test, we measured the concentration of C21 in forebrain tissue (*n* = 4–5). The brain concentration of C21 at 0.002 and 0.01 mg/g food were 14.2 ± 1.24 and 43.3 ± 5.58 nM, which was more than 25-fold higher than EC_50_ for hM3Dq (1.7 nM, Fig. 1f). These results suggest that long-term administration of C21 at 0.01 mg/g in the chow supplies sufficient agonist to stimulate the DREADD receptor in the brain, and we employed this dosage in the subsequent study.

### PVT–NAc activation by DREADD increased depressive-like episodes

**Fig. 2:**
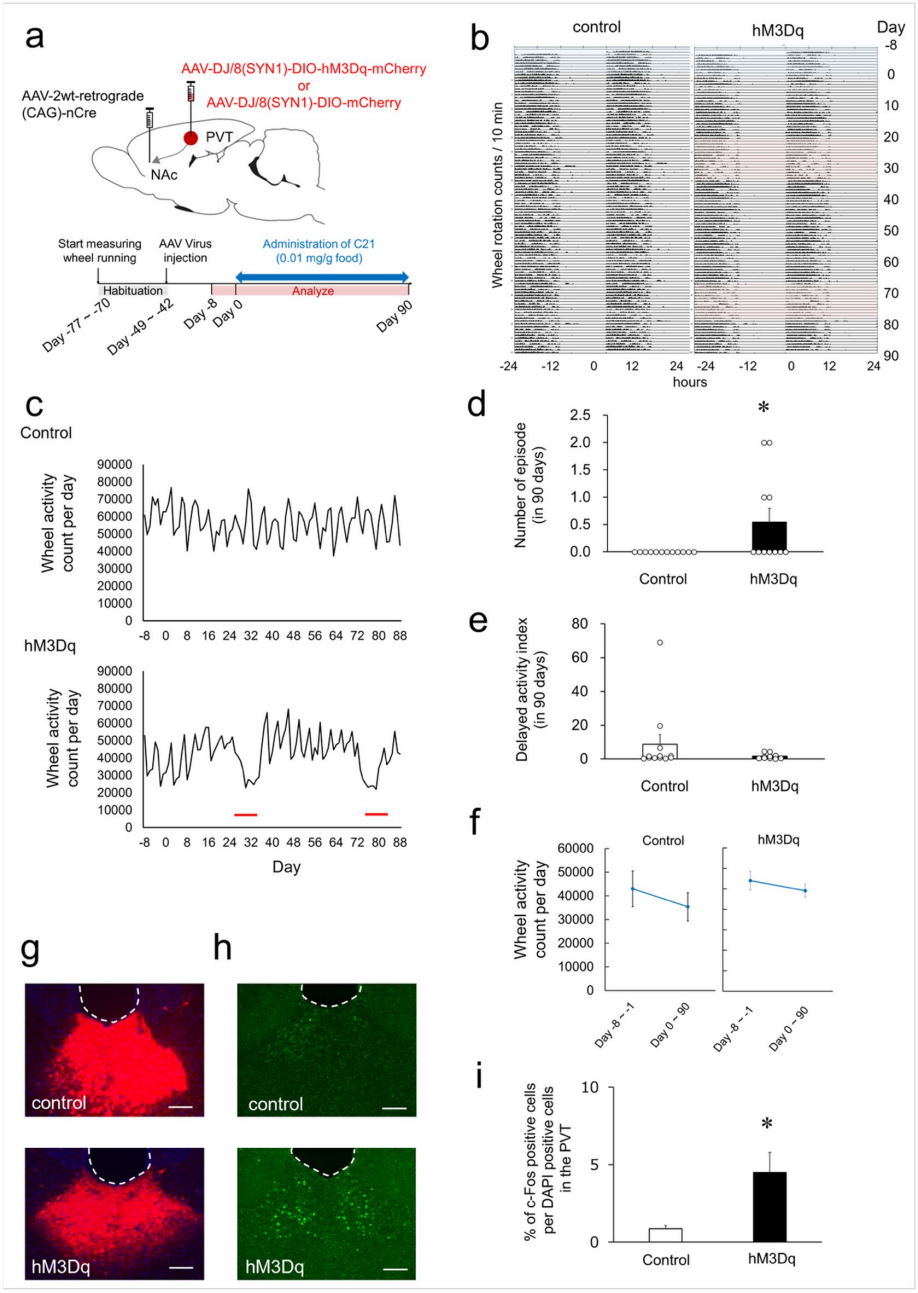
Impact of Activation of the PVT–NAc Neuronal Projection on Wheel-Running Activity (**a**) Schematic illustration of the viral strategy for expressing an excitatory DREADD (hM3Dq) or a control vector in the PVT–NAc circuit (upper), and the experimental timeline for virus injection and C21 administration (lower). (**b**) Double-plotted actograms showing wheel running activity of mice injected with AAV-Control (left) or AAV-hM3Dq (right) over a 98-day period. Each row is a bar graph of wheel rotation counts (each bar represents rotation counts per 10 min) for 48 h. Hatched in blue: before C21 administration, red: depressive-like episodes. (**c**) Daily wheel running counts were recorded from Day-8 to Day-1 (baseline period) and from Day 0 to Day 90 during C21 administration. The upper panel shows data from mice injected with a control vector, and the lower panel shows data from mice expressing hM3Dq. The horizontal axis illustrates the number of days from Day 0, the start of C21 administration, extending from Day-8 through Day 90. The vertical axis indicates the number of wheel movement per day, with thick lines highlighting depressive-like episodes. (**d**) Influence of activated neuronal pathway from the PVT to the NAc on depressive-like episodes in wheel-running activity. Each circle represents the number of depressive-like episodes of each mouse and each bar denotes the mean + S.E.M. over a 90-day period (control: *n* = 12, hM3Dq: *n* = 11). *p < 0.05 compared to the control group (Wilcoxon rank-sum test). (**e**) Effect of activated neuronal pathway from the PVT to the NAc on delayed activity index in wheel-running activity. Each circle represents delayed activity index of each mouse and each bar denotes the mean + S.E.M. over a 90-day period (control: *n* = 12, hM3Dq: *n* = 11). (**f**) Wheel-running activity before and after C21 administration. Parallel‑axis plots display the mean ± SEM values of wheel running counts per day measured before and after C21 administration. (**g**) Representative images showing mCherry expression in the PVT of mice injected with AAV-Control (mCherry) or AAV-hM3Dq (hM3Dq-mCherry fusion protein). The scale bar indicates 100 μm. (**h**) Representative images showing c-Fos expression in the PVT of mice injected with AAV-Control or AAV-hM3Dq. Scale bar indicates 100 μm. (**i**) The proportion of c-Fos positive cells in the PVT of mice injected with AAV-Control or AAV-hM3Dq. Each bar represents the mean + S.E.M. of the proportion of c-Fos positive cells in the PVT (*n* = 5). *p < 0.05 compared to the control group (Student’s t-test).

We examined the effect of DREADD-mediated activation of the PVT–NAc pathway on voluntary wheel-running activity, a measure of natural reward-seeking behavior in rodents^25,26^. Using a Cre-loxP system combined with retrograde AAV^27^, we selectively expressed the excitatory DREADD receptors (hM3Dq) in PVT neurons projecting to the NAc. To anatomically verify the PVT–NAc pathway, mice were injected with a DREADD (AAV-DJ/8(SYNI)-DIO-hM3Dq-L-mCherry) or a control virus (AAV-DJ/8(SYNI)-DIO-mCherry) into the PVT together with a retrograde Cre virus into bilateral NAc to target PVT neurons projecting NAc (Fig. 2a). After a recovery period from surgery (see Fig. 2a and Methods), mice received C21-containing food. Analysis of wheel-running activity during C21 administration revealed a significant increase in number of depressive-like episodes in DREADD virus-injected mice (0.5 ± 0.2) compared with controls (0.0 ± 0.0; *p* = 0.037) (Fig. 2b–d). No significant change in delayed activity index was observed between groups during C21 administration (8.7 ± 5.7 vs. 1.6 ± 0.5; *p* = 0.249) (Fig. 2e). In both groups, no significant difference in wheel-running activity was observed before and after C21 administration (Control group: 42,985 ± 7573 vs. 35,368 ± 5946; *p* = 0.437; DREADD group: 53,994 ± 4558 vs. 49,085 ± 3222; *p* = 0.390, Fig. 2f). Robust mCherry expression was observed in PVT neurons projecting to the NAc (Fig. 2g), and immunohistochemistry showed heightened c-Fos expression in the DREADD group (4.50 ± 1.30%) compared with control groups (0.86 ± 0.22%; *p* = 0.024, Fig. 2h, i), confirming activation of PVT neurons.

### Activation of PVT–BLA has no effect to wheel running behavior

**Fig. 3:**
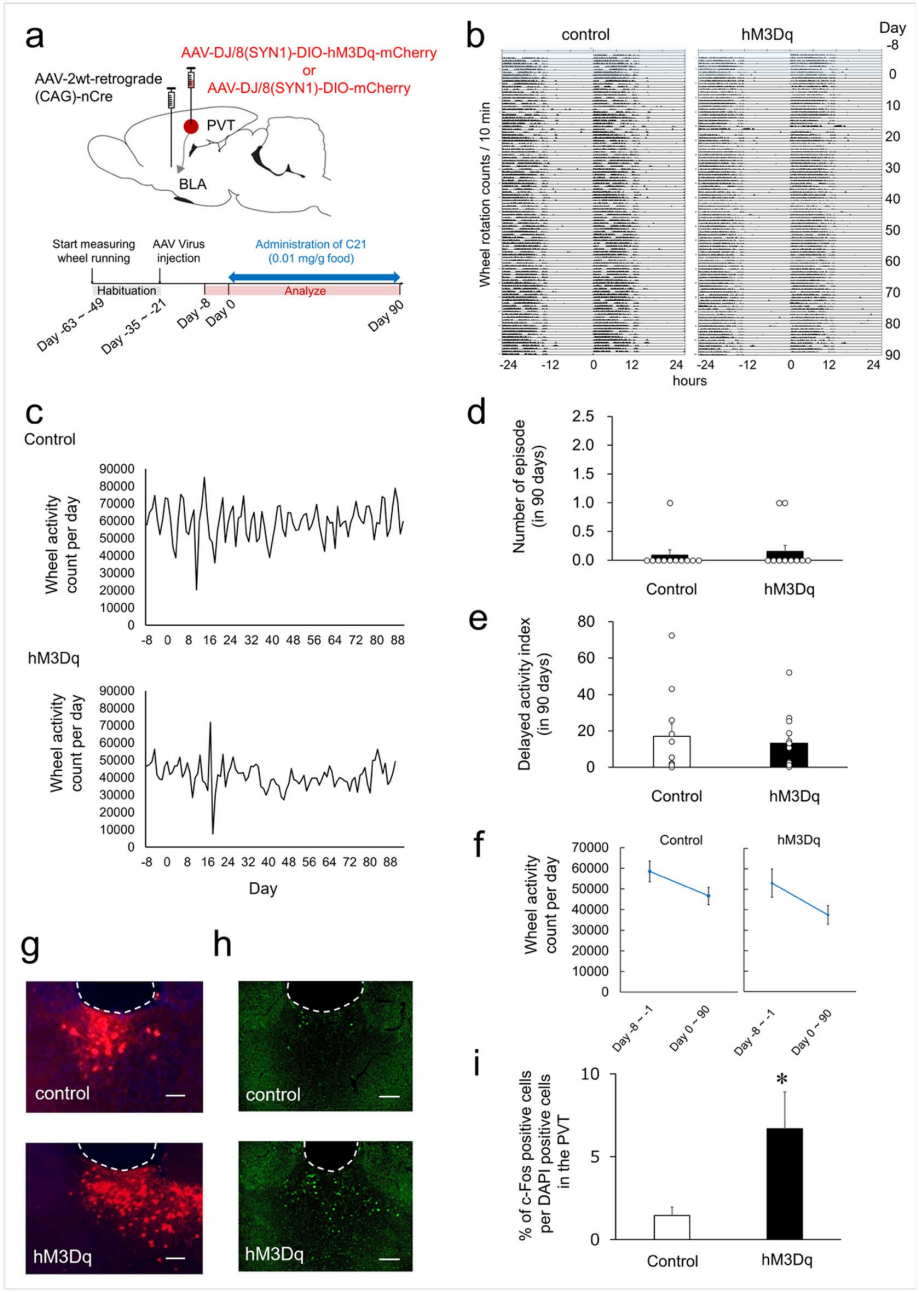
Impact of Activation in the PVT–BLA Neuronal Projection on Wheel-Running Activity (**a**) Schematic illustration of the viral strategy for expressing an excitatory DREADD (hM3Dq) or a control vector in the PVT–BLA circuit (upper), and the experimental timeline for virus injection and C21 administration (lower). (**b**) Double-plotted actograms showing wheel-running activity of mice injected with AAV-Control (left) or AAV-hM3Dq (right) over a 98-day period. (**c**) Daily wheel running counts were recorded from Day-8 to Day-1 (baseline period) and from Day 0 to Day 90 during C21 administration. The upper panel shows data from mice injected with a control vector, and the lower panel shows data from mice expressing hM3Dq. The horizontal axis indicates the number of days from Day 0, the start of C21 administration, extending from Day-8 through Day 90. The vertical axis reflects wheel rotation counts per day. (**d**) Effect of activated neuronal pathway from the PVT to the BLA on depressive-like episodes in wheel-running activity. Each circle represents the number of depressive-like episodes of each mouse and each bar shows the mean + S.E.M. over 90 days (control: *n* = 11, hM3Dq: *n* = 13). (**e**) Effect of activated neuronal pathway from the PVT to the BLA on delayed activity index in wheel-running activity. Each circle indicates delayed activity index of each mouse, and each bar signifies the mean + S.E.M. of delayed activity index over a 90-day period (control: *n* = 11, hM3Dq: *n* = 13). (**f**) Wheel-running activity before and after C21 administration. Parallel‑axis plots display the mean ± SEM values of wheel running counts per day measured before and after C21 administration. (**g**) Representative images depicting mCherry expression in the PVT of mice injected with AAV-Control or AAV-hM3Dq. Scale bar denotes 100 μm. (**h**) Representative images of c-Fos expression in the PVT of mice injected with AAV-Control or AAV-hM3Dq. Scale bar indicates 100 μm. (**i**) The proportion of c-Fos positive cells in the PVT of mice injected with AAV-Control or AAV-hM3Dq. Each bar represents the mean + S.E.M. of the proportion c-Fos positive cells in the PVT (control: *n* = 5, hM3Dq: *n* = 6). *p < 0.05 compared to the control group (Student’s t-test).

Similarly, we next assessed the effect of activating PVT neurons projecting to the BLA via DREADD on wheel-running activity. Following habituation, either the DREADD or control virus was injected into the PVT, and the retrograde Cre was injected into the BLA to target PVT–BLA projections (Fig. 3a). After recovery from surgery (see Fig. 3a and “Methods” section), mice received C21 (0.01 mg/g food), and activity was measured over several weeks. No significant differences were observed between the DREADD- and control virus-injected group in the number of depressive-like episodes (0.2 ± 0.1 vs. 0.1 ± 0.1; *p* = 1.000) or the delayed activity index during C21 administration (17.0 ± 6.9 vs. 13.3 ± 4.2; *p* = 0.634) (Fig. 3b–e). In both groups, no significant difference in wheel-running activity was observed before and after C21 administration (Control group: 58,470 ± 5030 vs. 46,627 ± 4335; *p* = 0.087; DREADD group: 53,053 ± 6891 vs. 37,478 ± 4571; *p* = 0.072, Fig. 3f). The mCherry fluorescence was detected in the PVT (Fig. 3g). Immunohistochemical analysis revealed significantly elevated expression in the PVT of the DREADD group (6.68 ± 2.22%) compared with control group (1.45 ± 0.50%; *p* = 0.048), supporting successful target activation (Fig. 3h, i).

### Largely segregated projections from PVT to the NAc and BLA

**Fig. 4:**
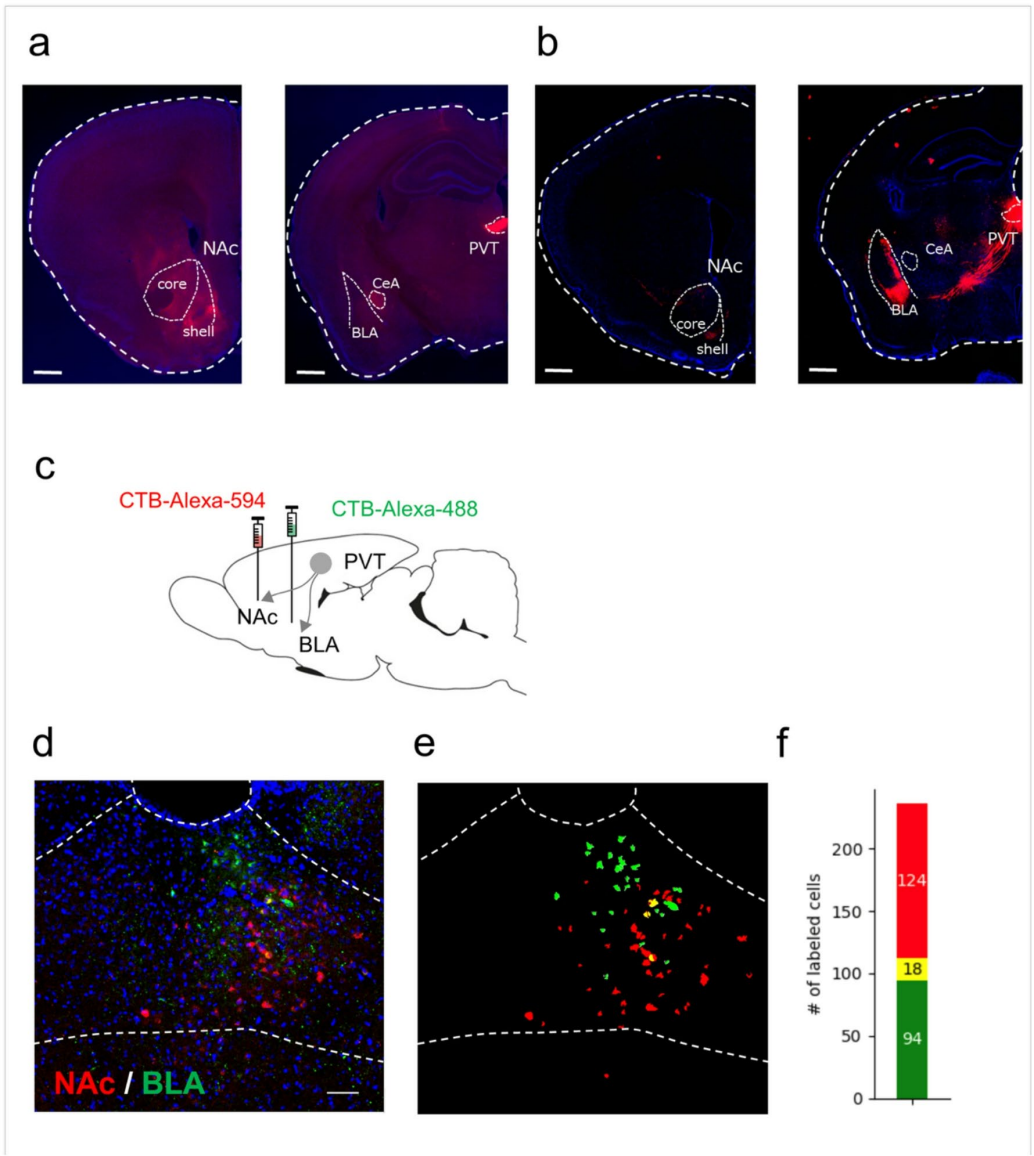
Collateralization of Projections from the PVT to the NAc and Amygdala (**a**) AAV-Control in the PVT–NAc pathway. Scale bar equals 500 μm. (**b**) Representative image of mCherry expression in the NAc and amygdala of mice injected with AAV-Control in the PVT–BLA pathway. Scale bar: 500 μm. (**c**) Schematic illustration of tracer injections into the NAc and BLA and retrograde labeling in the PVT. (**d**) PVT (delineated by dotted lines) neurons are retrogradely labeled by unilateral injections of CTB-alexa 594 (red) at NAc and CTB-alexa 488 at BLA (green). Blue: DAPI, scale bar: 100 μm. (**e**) Segmentation of labeled areas by CTB-Alexa 488 (green) and CTB-Alexa 594 (red). (**f**) A bar graph for the number of cells labeled by Alexa-488 (green), Alexa-594 (red), and both dyes (yellow, 8 sections from 3 mice).

To examine degree of overlaps between PVT–NAc and PVT–BLA neurons, we compared the density of mCherry-labeled axon terimnals in NAc and BLA post-wheel activity observation. In mice expressing mCherry in the PVT–NAc projections, dense virus-labeled axonal terminals were found in the NAc but few in the BLA. In addition to the NAc, mCherry-positive fibers were observed in the central amygdala (CeA) (Fig. 4a). Conversely, in mice expressing mCherry in the PVT–BLA neurons, strong signals of labeled axonal were obserbed in the BLA but scarcely in the NAc or CeA (Fig. 4b). Furthermore, retrograde labeling of axons from NAc (injected Cholera Toxin subunit B (CTB)-Alexa 594) and BLA (injected with CTB-Alexa 488, Fig. 4c) showed majority of neurons are labeled by one of the dyes in PVT (Fig. 4d–f). These findings indicate that PVT-BLA and PVT-NAc are largely segregated population and thus support that selective activation of the PVT–NAc pathway may trigger depressive episodes.

## Discussion

In this study, we investigated the neural circuit basis of the depressive-like episode by chemogenetic activation of subset of PVT neurons. We verified the use of DREADD agonist C21 for long-term behavioral analysis by searching for potential off-targets and accurately measuring its concentration of the compound in the brain. Activation of PVT neurons projecting to the NAc induced depressive-like episodes in wheel-running activity, whereas activation of those projecting to the BLA had no such effect, suggesting that the PVT–NAc pathway plays a crucial role in the regulation of depressive-like behavior, potentially relevant to depressive episodes in bipolar disorder.

To ensure the specificity of C21, we examined its off-target profile and its dosages for chronic administration. Previous work shows that C21 has high affinity for hM3Dq receptors, with an EC_50_ of 1.7 nM^22^. However, our screening results revealed that C21 at 100 nM inhibited over 50% of binding at several off-target sites, including histamine H_1_ and serotonin 5-HT_2_ receptors, which are implicated in sedation and emotional regulation^28–33^. Importantly, acute C21 administration does not affect locomotion^34^, and our chronic in-feed administration in mice did not alter wheel-running activity, indicating minimal side effects during long-term behavioral analyses.

The PVT projects to multiple brain regions involved in anxiety and motivated behavior, including the NAc, amygdala, and medial prefrontal cortex. Prior studies have established that chronic activation of PVT neurons increases depressive-like episodes in wheel-running activity in mice, but the mechanisms underlying how specific circuits contribute to the emergence of these episodes remain to be elucidated. We found that chemogenetic activation of PVT-NAc, but not PVT-BLA, induced depressive-like episodes. Given that wheel running is naturally rewarding for rodents, our findings indicate chronic activation of PVT would alter functions of NAc-mediated reward system. Whether chronic activation of PVT affect to other depression-related behaviors such as anxiety, which is primarily regulated by the amygdala, remains to be elucidated.

The NAc is predominantly composed of medium spiny neurons (MSNs), which express either dopamine D_1_ or D_2_ receptors (D1-MSNs and D2-MSNs). Stimulation of D1-MSNs, but not D2-MSNs, alleviates depressive behavior, suggesting that these neuronal subtypes exert distinct effects on mood regulation^35^. The long-term activation of PVT–NAc may disrupt the balance of these neuronal populations. Previous studies have suggested that PVT–CeA pathway regulates behaviors associated with depression and fear^10,36^. In our experiments, axonal terminals from PVT neurons projecting to the NAc were also observed in the CeA^37^, indicating that the involvement of the PVT–CeA pathway cannot be excluded, although our findings suggest the PVT–NAc circuit plays a key role in depressive-like episodes. Conversely, activation of PVT neurons projecting to the BLA did not induce depressive-like episodes. This differential impact may arise from the distinct functional roles of the PVT–NAc and PVT–BLA pathways.

In summary, this study identified the PVT–NAc pathway as a critical neural circuit linked to the induction of depressive-like episodes in the wheel-running activity of mice, providing insights into circuit-level mechanisms relevant to depressive episodes in bipolar disorder. These results may shed light on neural circuitry-related abnormalities specific to depressive episodes in bipolar disorder. Future research should aim to identify and characterize specific molecular modulators of the PVT–NAc circuit, which could pave the way for developing novel therapeutic interventions targeting depressive episodes in bipolar disorder.

## Limitation of this study

Several limitations of our study should be acknowledged. While we demonstrated the role of PVT-NAc role in the expression of recurrent depressive-like episodes represented by decrease of wheel running activities, depressive behaviors were not comprehensively characterized across multiple experimental paradigms. Future studies employing additional behavioral assays, such as the forced swim test and sucrose preference test, as previously applied in Polg tg mice^20^, would strengthen the behavioral phenotyping. Additionally, our study exclusively utilized female mice, as Polg tg females exhibit pronounced depressive-like episodes. It remains to be determined whether PVT-NAc activation similarly influences mood-related behaviors in male mice, which warrants further investigation. Another important aspect not addressed in this study is the impact of PVT activation on appetite regulation and food consumption rates, which could provide insights into additional behavioral dimensions influenced by this circuitry. Finally, we did not differentiate between the functional contributions of PVT-NAc versus PVT-CeA pathways. Dissecting these distinct circuitries in future studies will be crucial to fully elucidate the role of PVT projections in depressive-like states.

## Methods

### Animals

Animal experiments were approved by Institutional Animal Care and Use Committee of Sumitomo Pharma (approval number: AN13914, AN14223, AN14583, AN14790, 24RA0026) and performed in accordance with the approved protocols and the regulations for animal experiments and related activities of the institution. All methods were carried out in accordance with ARRIVE guidelines. We used female C57BL6/JJcl mice (CLEA Japan, Tokyo, Japan) in this study. All animals were obtained at 7–8 weeks of age, with body weights ranging from 16.7 to 21.5 g upon arrival. All animals were housed in controlled 12:12 light–dark cycles and had access to diet and water ad libitum. All animal experiments were Animal Care and Use Committee of Sumitomo Pharma. For euthanasia, we placed mice in a chamber dispensed carbon dioxide gas at 30–70% flow rate until respiratory arrest (about 5 min) and euthanized by cervical dislocation.

### Viral vector production

To selectively stimulate PVT neurons, we employed the DREADDs^27,38,39^ in combination with Cre-dependent double-floxed inverted open reading frame (DIO)^40^ and retrograde AAV serotype^27^. All plasmids and AAVs used in this study were prepared in-house, unless otherwise noted. We transfected 3 plasmids, a helper plasmid pHelper (Agilent, Santa Clara, CA), a rep/cap plasmid, and an AAV transfer plasmid into HEK293 cells. For Cre-dependent DIO viruses, we used pAAV-DJ/8 (Cell Biolabs, San Diego, CA) as a rep/cap plasmid, together with pAAV-Syn-DIO-hM3Dq for AAV-DJ/8(SYNI)-DIO-mCherry and pAAV-Syn-DIO-mCherry with AAV-DJ/8(SYNI)-DIO-hM3Dq-mCherry. For Cre-expressing retrograde viruses, we used pAAV-2-retrograde as a rep/cap plasmid together with pAAV-CAG-nCre. Viral vectors were stored at − 80℃, and the genomic titer of each virus was determined using quantitative PCR. The titer of viruses was as follows; AAV-DJ/8(SYNI)-DIO-hM3Dq-mCherry: 1 × 10^13^ VG/mL, AAV-DJ/8(SYNI)-DIO-mCherry: 1 × 10^13^ VG/mL, and AAV-retrograde (CAG)-nCre: 4 × 10^13^ VG/mL.

### Stereotaxic injection

Mice (11–16 weeks old) were anesthetized with isoflurane, and both the skin and the scalp were disinfected with a 10% povidone-iodine solution (Shionogi Pharma, Osaka, Japan). AAV particles were injected into following coordinates (in mm): PVT (AP − 1.75, ML 0.0, and DV 3.05), NAc (AP 1.4, ML ± 0.8, and DV 4.3), and BLA (AP − 1.4, ML ± 3.3, and DV 4.8). The AP and ML coordinates were defined relative to the bregma, and the DV was determined relative to the brain surface. We injected virus solution at a rate of 0.1 μL/min for 2 min (a total of 0.2 μL/injection site) using a 2 μL Neuros syringe with a 33-gauge needle (Hamilton Company, NV, USA) and a motorized stereotaxic microinjector (NARISHIGE Group, Tokyo, Japan). After injection, the needle was left in place for 5 min to facilitate diffusion of the solution. After the mice recovered from anesthesia, they were returned to their wheel-running cages. Both Alexa 488 and 594 conjugated cholera toxin B subunit (CTB-Alexa 488: C34775 and CTB-Alexa 594: C34777, ThermoFisher Scientific) were diluted at 10 mg/ml in PBS, and 100 nl each of solutions was injected at NAc (CTB-Alexa 594) and BLA (CTB-Alexa 488). Injected animals were analyzed 1 week after injection.

### Chemical reagents

C21^22^ was synthesized in our laboratories. The compound was mixed with powdered diet CE-2 (CLEA Japan, Tokyo, Japan) at 0.002 or 0.01 mg/g and provided ad libitum in a specialized feeding container (SHINANO manufacturing, Tokyo, Japan).

### Radioligand binding assay

Radioligand binding assays for 39 targets (receptors, ion channels and transporters) were conducted using standard techniques at Eurofins Panlabs Discovery Services Taiwan, Ltd. (Taipei, Taiwan). Assay procedures were based on those described in Eurofins Panlabs Taiwan Protocols (https://apac.eurofinsdiscovery.com/), as summarized in Table 1. The assay was carried out in duplicate.

### Pharmacokinetic parameter of DREADD agonist C21

Mouse forebrains were collected after perfusion with PBS under isoflurane anesthesia. The brains were frozen on dry ice and stored at − 80℃ until use. The brains were homogenized with 4 times weight PBS. Fifty microliters of brain homogenate solutions were added to 10 μL of methanol and 200 μL of methanol containing internal standard (1 μM Phenytoin). The mix solutions were kept in a freezer for more than 30 min before centrifugation at 1800 × g for 10 min at 4 °C. One-hundred microliters of supernatants were diluted by 200 μL of water, centrifuged at ca. 1800 × g for 5 min at 4 °C and applied to a liquid chromatography-tandem mass spectrometry. Analyses were carried out using API-4000 (AB Sciex, Framingham, MA, USA) with the electrospray ionization interface in positive ion mode. A LC-20A Series high performance liquid chromatography (HPLC) system (Shimadzu, Kyoto, Japan) was used with an analytical column (Unison UK-C18, 3 μm, 20 × 2.0 mm i.d.; Imtakt Corporation, Kyoto, Japan).

### Long-term analysis of wheel running

Wheel-running activity of the female mice was recorded and analyzed as described previously^19^. Each mouse was housed in a cage with a running wheel (5 cm width, 14 cm diameter) and an automatic counter for wheel rotation (O’hara & Co., Ltd., Tokyo, Japan). In the habituation period prior to administration or virus injection, mice with abnormal running patterns, defined as less than 60,000 counts per day or the delayed activity index over 3, were excluded from behavioral analyses. The delayed activity index was calculated as a percent of the activity counts during the first 3 h of the light period with the total activity counts during the previous dark period (12 h) as described previously^19^. After virus injection surgeries, we transferred animals to regular cages and wait until all of injected animals were recovered (4–6 weeks for PVT-NAc experiment and 3–4 weeks for PVT-BLA experiment. See Figs. 2a, 3a). Their wheel running activities were monitored for 8 days with regular chows then for another 90 days with C21-containing diet. Depressive-like episodes were defined by the relative strength index (RSI) and reverse RSI as reported previously^20^. We used the modified criteria (minimum 7 days with RSI < 50, at least 1 day with RSI < 30, minimum 7 days with reverse RSI < 50, and at least 1 day with reverse RSI < 30) to define the period of a depressive-like episode. Episodes coinciding with the start of DREADD agonist C21 administration were excluded if the pre-treatment RSI was below 50.

### Histochemistry

Mice were deeply anesthetized with isoflurane and perfused with PBS followed by 4% paraformaldehyde (FUJIFILM Wako Pure Chemical, Osaka, Japan). Brains were removed and immersed in 4% paraformaldehyde at 4 °C overnight, followed by immersion in 30% (w/v) sucrose solution at 4 °C for 24 h. The fixed brains were embedded into the O.C.T. compound (Sakura Finetek Japan, Tokyo, Japan) and sectioned at 30 μm thickness with a cryostat (Leica Biosystems, Nussloch, Germany).

The free-floating sections were collected in PBS and stored at − 80 °C until use. Before staining, Free-floating sections were rinsed with 0.1% (v/v) Triton X-100 in PBS, and then blocked with 10% (v/v) Donkey serum, 2% BSA, and 0.3% (v/v) Triton X-100 in PBS. Sections were incubated with primary antibodies against c-Fos (1:500, #2250, Cell Signaling Technology, Massachusetts, USA) overnight at 4 °C. After washing with PBS for three times, sections were incubated with an Alexa488-conjugated anti-rabbit IgG (1:200; ab150073, Abcam) for 2 h at room temperature. Sections were washed three times for 5 min with PBS and covered with HardSet Antifade Mounting Medium with DAPI (Vector Laboratories, Newark, USA) on slides. Sections were imaged with a BZ-710 Viewer (KEYENCE, Osaka, Japan). The percentage of c-Fos positive cells in the PVT was calculated using ImageJ (Fiji) as the ratio o1f. c-Fos–positive cells to the total number of DAPI-stained nuclei. Sections of CTB-injected animals were stained with DAPI and imaged with Leica SP8 confocal microscope. ImageJ’ Particle analyzer function was used for segmentation of CTB-labeled areas. Particles adjacent to or overlap with DAPI signals were counted as cells.

### Data analysis and statistics

Statistical analysis was performed using Microsoft Excel (Microsoft Japan, Tokyo, Japan) and Stat Preclinica/SAS (Takumi Information Technology Inc., Tokyo, Japan). To assess dose-dependent effect of C21, one-way ANOVA was performed. The Wilcoxon rank-sum test was used to evaluate differences in the number of depressive-like episodes between two groups, while paired Student’s t test was applied to compare differences in the delayed activity index and the number of c-Fos-positive cells in the PVT between two groups and wheel running counts per day measured before and after C21 administration. Differences were considered statistically significant at *p* < 0.05.

## Data Availability

The datasets generated during and/or analyzed during the current study are available from the corresponding author on reasonable request.
